# The effects of climate and catchment characteristic change on streamflow in a typical tributary of the Yellow River

**DOI:** 10.1038/s41598-019-51115-x

**Published:** 2019-10-10

**Authors:** Xizhi Lv, Zhongguo Zuo, Yongxin Ni, Juan Sun, Henian Wang

**Affiliations:** 1 Yellow River Institute of Hydraulic Research, Key Laboratory of the Loess Plateau Soil Erosion and Water Loss Process and Control of Ministry of Water Resources, Zhengzhou, 450003 China; 2 Institute of Wetland Research, Chinese academy of forestry, Beijing Key Laboratory of Wetland Services and Restoration, Beijing, 100091 China

**Keywords:** Environmental health, Phenology, Hydrology

## Abstract

Hydrological cycle changes that occur due to a changing environment is a hot topic in the field of hydrological science. It is of great practical significance to study the response mechanism of hydrological process change for future water resources planning and management. In this study, the effects of climate and watershed characteristic change on the streamflow in a typical tributary of the Yellow River (the Fen River watershed) are studied based on the Budyko hypothesis. The results show that: the sensitivity coefficients of streamflow to precipitation, potential evapotranspiration, and the watershed characteristic coefficient were 0.1809, −0.0551, and −27.0882, respectively. This meant that a 1 mm decrease in the precipitation would induce a 0.1809 mm decrease in the streamflow. Additionally, a 1 mm decrease in the potential evapotranspiration would induce a 0.0551 mm increase in the streamflow, and an increase of 1 in the watershed characteristic coefficient would induce a 27.0882 mm decrease in the streamflow. The streamflow of the Fen River watershed showed a significant decreasing trend during the reference period (1951–1977). In addition, the streamflow of the change period (1978–2010) decreased 26.87 mm; and this was primarily caused by watershed characteristic change which accounted for 92.27%, while climate change only accounted for 6.50%.

## Introduction

How the hydrological cycle responds in a changing environment is an important area of investigation in hydrological science^1^. Numerous studies have been conducted to determine how different factors impact streamflow generation^2–4^. Many hydrological models have been developed to explore streamflow processes. However, model and parameter calibrations have remained underdeveloped. It is necessary to have parameter and model certainty, and this uncertainty has led to inaccurate simulation results^5,6^. More and more researchers have begun to use climate sensitivity method to study the effects of climate change on watershed streamflow^7–9^. A sensitivity analysis can provide crucial information regarding climate change. Climate sensitivity methods are convenient methods to research the relationship between climate factors and watershed streamflow, and they have also led to the discovery of a new version of this relationship^10^. Most sensitivity analyses have been based on theoretical models. Among all of the models, the hydrothermal coupled equilibrium equation has been the most widely used model by researchers. The equation, based on the Budyko hypothesis, is a typical representative model used to explore hydrothermal coupled equilibrium^11,12^. This model was used by Chinese researchers for both humid and non-humid areas, and the results indicated the applicability of this model in China.

Climate change and the underlying surface of the watershed are the two dominate factors that impact streamflow change. The impact of climate change on the hydrological cycle will be a change in the global hydrology distribution. Of all of the climate factors, precipitation and potential evapotranspiration are the most important for the determination of climate characteristics^13^. There are many factors that influence the underlying surface characteristics of a watershed, including land use change, hydraulic engineering, water resources development, and others. The underlying surface condition of a watershed has been reported to have a more important function than climate change on the hydrological cycle, and it contributed more than 50% to streamflow change^14^.

Numerous methods have been proposed to distinguish the impacts of climate and catchment variability on water yield. Along with “bottom-up” experimental approaches, energy-based theoretical equations that describe the climate and water balance have been developed and applied in what is often called a “top-down” approach^15–17^. Among them, the framework developed by Budyko (1974)^18^ has received the most attention and application. Based on the framework, the elasticity method initially proposed by Schaake (1990)^19^ uses elasticity coefficients to assess the sensitivity of the water yield to climate factors, and this method has been improving as a result of the efforts of other researchers^20^. Many previous studies^21–24^ have used the climate elasticity method to quantify the effects of climate and catchment variability on streamflow and to analyze the main causes of streamflow changes.

The phenomenon of runoff streamflow decrease has attracted wide attention during the past 50 years, especially for government decision makers. The Yellow River is the second longest river in China, and its water resources have significantly decreased. The Fen River, as the second largest tributary in China, is crucial for water resource management in the future. The objectives of this study are to (1) analyze the changes in climate, streamflow and catchment characteristics; (2) calculate the sensitivities of streamflow to climate and catchment changes; and (3) identify the contributions of climate and catchment variability to streamflow changes in the Fen River watershed.

## Materials and Methods

### Study area

**Figure 1:**
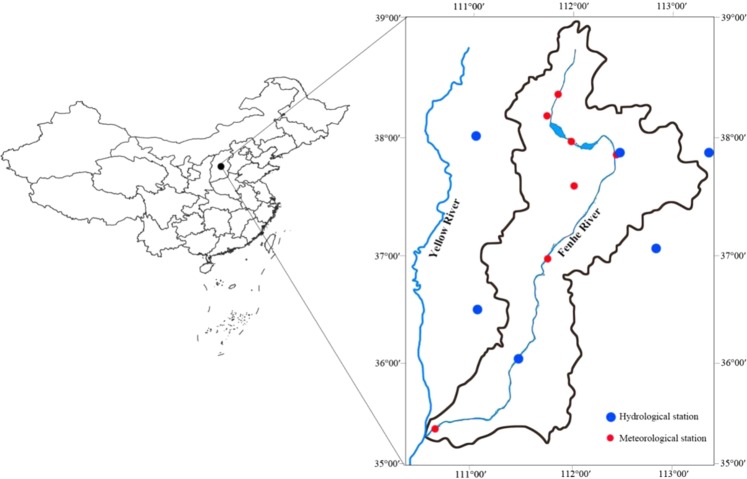
The location of the Fen River watershed, hydrological stations and meteorological stations.

The Fen River is the second longest tributary in the Yellow River watershed (Fig. 1). Its main stream is 694 km long, and the area of the watershed is 39,471 km^2^ (35.3°–39.0°N, 110.5°–113.5°E). The Fen River watershed is primarily dominated by mountains, and hilly mountain areas account for more than 70% of the total area. It is bounded by Taihang Mountain to the east and Lvliang Mountain to the west. The highest elevation of the area is 2,786 m in the north and the lowest is 240 m in the south. Climate in this watershed is a warm temperate continental monsoon condition. The annual mean temperature is 6.2~12.8 °C, annual mean precipitation is 434~528 mm, and approximately 72% of the precipitation falls between June and September. The landforms are usually capped by a thick layer of loess due to dust deposition during the Quintenary, according to the FAO-90. Soil types in the basin are Calcaric Cambisols and Calcaric Fluvisols, which are highly alkaline.

### The Budyko hypothesis

Budyko (1974)^18^ reported that actual evapotranspiration (E) was determined by water supply (precipitation) and evaporation capacity (net radiation or potential evapotranspiration), E can be calculated by formula:

1 $$\frac{E}{P}=f({E}_{0}/P)$$

where *E* is the actual evapotranspiration amount in this watershed (mm); *P* is precipitation (mm); *E*_0_ is potential evapotranspiration (mm).

Based on the Budyko hypothesis, Baopu Fu put forward a group of differential equations regarding the Budyko hypothesis, and rewrote the equation as:

2 $$\frac{E}{P}=1+\frac{{E}_{0}}{P}-{[1+{(\frac{{E}_{0}}{P})}^{\omega }]}^{1/\omega }$$

where *ω* is a parameter of the underlaying characteristic that relates to land use condition, vegetation, soils, and other characteristics^25–27^.

Therefore, the Budyko hypothesis was developed to be a synthetic expression that considers the watershed characteristic as well, and it is expressed as the following:

3 $$\frac{E}{P}=f(\frac{{E}_{0}}{P},\omega )$$

### Watershed streamflow sensitivity

A similar parameter was developed to represent the sensitivity inferred from the elasticity coefficient^19^. This parameter can reflect the streamflow change amount caused by a unit change in the dependent variable (*∂Q/∂X*).

Based on the long-term water balance equation (*P* = *E* + *Q*), Eq. (2) was used to deduce the simulation equation of streamflow (*Q*), shown as:

4 $$Q={[{P}^{\omega }+{{E}_{0}}^{\omega }]}^{1/\omega }-{E}_{0}$$

Then, using calculus, the partial derivative result of Eq. (4) is the sensitivity parameter of *Q* to *P*, *E*_0_ and *ω*, as follows:

5 $$\frac{\partial Q}{\partial P}={[1+{(\frac{{E}_{0}}{P})}^{\omega }]}^{(1/\omega -1)}$$

6 $$\frac{\partial Q}{\partial {E}_{0}}={[1+{(\frac{P}{{E}_{0}})}^{\omega }]}^{(1/\omega -1)}-1$$

7 $$\frac{\partial Q}{\partial \omega }={[{P}^{\omega }+{{E}_{0}}^{\omega }]}^{1/\omega }\cdot [(-\frac{1}{{\omega }^{2}})\cdot \,\mathrm{ln}({P}^{\omega }+{{E}_{0}}^{\omega })+\frac{1}{\omega }\cdot \frac{1}{{P}^{\omega }+{{E}_{0}}^{\omega }}\cdot (\mathrm{ln}\,P\cdot {P}^{\omega }+\,\mathrm{ln}\,{E}_{0}\cdot {{E}_{0}}^{\omega })]$$

where *ω* is calculated using the least squares method.

### The Mann-Kendall test

 

1. The Mann-Kendall trend test
  The Mann-Kendall trend test method is one of the most widely used non-parametric tests for the detection of varying trends in climatic or hydrological data in a time series. It is based on the statistic *S*:
  8 $$S=\mathop{\sum }\limits_{i=1}^{n-1}\mathop{\sum }\limits_{j=i+1}^{n}{sgn}({x}_{j}-{x}_{i})$$
  9 $${sgn}(x)=\{\begin{array}{ccc}1, & if & {x}_{j}-{x}_{i} > 0\\ 0, & if & {x}_{j}-{x}_{i}=0\\ -1, & if & {x}_{j}-{x}_{i} < 0\end{array}$$
  where *x*_*i*_ and *x*_*j*_ are two simple values of the sequential time series data (*x*_1_, …, …, *x*_*n*_); and *n* is the length of the data set.
  The variance associated with statistic *S* (*Var(S)*) is calculated as:
  10 $$Var(S)=\frac{n(n-1)(2n+5)}{18}$$
  Then, the test statistic *Z* can be calculated as follows:
  11 $$Z=\{\begin{array}{ccc}\frac{S-1}{\sqrt{Var(S)}}, & if & S > 0\\ 0 & if & S=0\\ \frac{S+1}{\sqrt{Var(S)}}, & if & S < 0\end{array}$$
  The presence of a statistically significant trend is evaluated using the *Z* value. A positive (negative) value of *Z* indicates an upward (downward) trend. In addition, the change trend of the data set is significant at the level of *α* if |*Z*| ≥ *Z*_*1-α/2*_, where *Z*_*1-α/2*_ is obtained from the standard normal cumulative distribution Tables.
2. The Mann-Kendall change point test

The test statistic *S*_*k*_ is defined as follows:

12 $${S}_{k}=\begin{array}{cc}\mathop{\sum }\limits_{i=1}^{k}\mathop{\sum }\limits_{j=1}^{i-1}{\alpha }_{ij} & (k=2,3,4,\cdots ,n)\end{array}$$

13 $${\alpha }_{ij}=\begin{array}{ccc}\{\begin{array}{c}1\\ 0\end{array} & \begin{array}{c}{x}_{i} > {x}_{j}\\ {x}_{i}\le {x}_{j}\end{array} & 1\le j\le i\end{array}$$

and the statistic index *UF*_*k*_ is defined as follows:

14 $$U{F}_{k}=\begin{array}{cc}\frac{{S}_{k}-E({S}_{k})}{\sqrt{Var({S}_{k})}} & k=1,2,3,\cdots ,n\end{array}$$

where:

15 $$E({S}_{k})=\frac{k(k-1)}{4}$$

16 $$Var({S}_{k})=\frac{k(k-1)(2k+5)}{72}$$

A backward sequence *UB*_*k*_ is calculated using the same equation but with a reversed series of data. If there were a match point between the two curves (*UF*_*k*_ and *UB*_*k*_), then the match point would be regarded as the change point.

### Data

Hydrological process data from 1951 to 2010 were collected from China Hydrology Yearbook. Meteorological data were collected from four weather stations that are located near the research area. These data were also collected for the years from 1951 to 2010. The daily data included the mean temperature, highest temperature, mean relative humidity, sunshine hours, precipitation, and mean wind speed.

### Framework presentation

**Figure 2:**
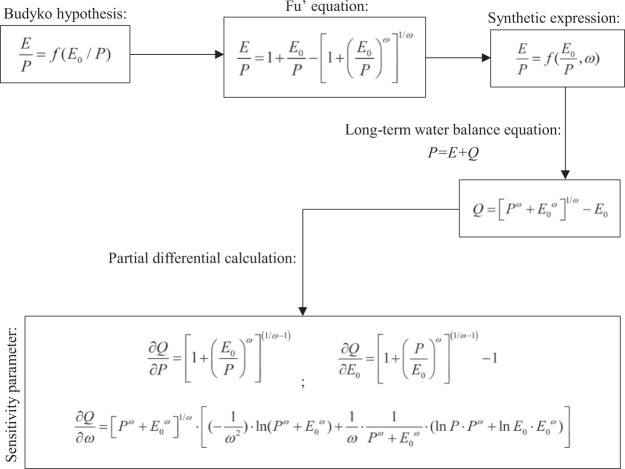
The graphical presentation of the framework in this study.

To better describe the research process, we develop a graphical presentation of the framework, seeing in Fig. 2.

## Results

### Hydrological variations in Fen River watershed

**Figure 3:**
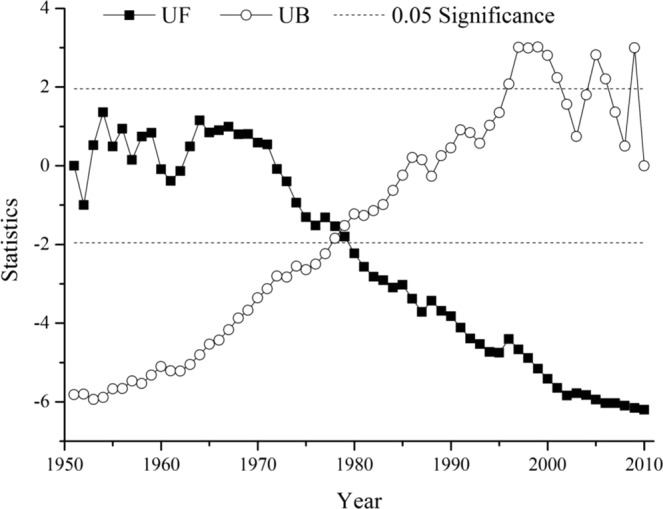
Mann-Kendall mutation test of annul streamflow amount in the Fen River watershed.

The Mann-Kendall trend test was performed annually from 1951 to 2010 for the Fen River watershed. The statistical test value of the Mann-Kendall test was −5.82, and the absolute value (|Z|) was higher than 2.58. The statistical test value shows that there was a significant decreasing trend in the annual streamflow amount, and the significance level was 0.01. Simultaneously, the Mann-Kendall mutation test was conducted for the Fen River watershed. The UF and UB values were used to represent the results from the Mann-Kendall mutation test (Fig. 3). The critical values of the UF and UB were 1.96 and −1.96, respectively, with a confidence level of 0.05. The UF and UB values in this study were both more than the critical value, and their lines crossed in 1978. Moreover, the intersection was between these two critical lines. Therefore, a streamflow mutation occurred in 1978.

**Figure 4:**
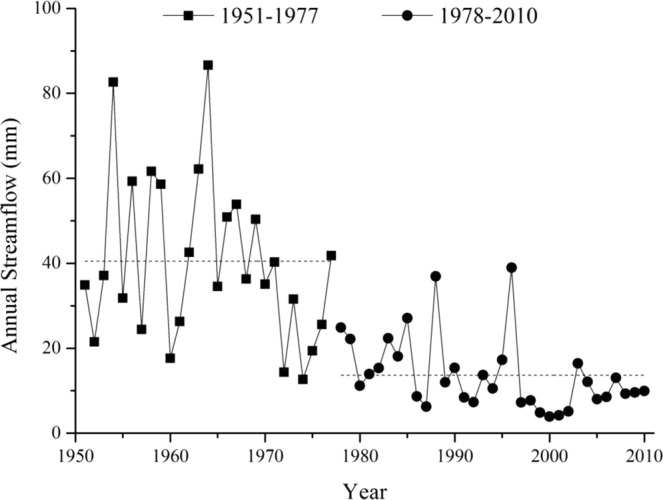
Annual streamflow comparison of reference period and change period in the Fen River watershed.

Due to the mutation point that occurred in 1978, the research period was divided in two stages: a reference period from 1951 to 1977 and a change period from 1978 to 2010. The annual streamflow amounts for the Fen River watershed are shown in Fig. 4. Figure 4 shows the mean annual streamflow of reference period was 40.51 mm, and it was 13.64 during the change period. The annual streamflow decreased 26.87 (66.33%).

### Effects of climate change on streamflow in the Fen River watershed

Based on annual data of the Fen River watershed from 1951 to 2010, the mean streamflow in this area was 25.74 mm. The mean annual precipitation was 429.18 mm, and the mean long-term potential evapotranspiration was 942.31 mm. These data were used in Eq. (4), and the watershed characteristic parameter was 3.1022 for the Fen River watershed. Then Eqs (5,6) and (7) were combined, and the sensitivity coefficient was calculated to be *∂Q/∂E*_0_ = −0.0551. This means that 1 mm of precipitation increase will increase runoff by 0.1809 mm; and a 1 mm potential evapotranspiration increase will decrease runoff by 0.0551 mm. The climate characteristics for the two periods in the Fen River watershed are shown in Table 1.

**Table 1:** Climate characteristics of two periods in Fenhe watershed.

Period	P/mm	E_0_/mm
1951–1977	438.51	955.11
1978–2010	421.54	931.84
Change/Δ	−16.57	−23.28

Based on the sensitivity coefficient and climate change amount of the streamflow in the Fen River watershed, the impact of climate change on the streamflow amount was obtained as:

$${\Delta }{Q}_{climate}=\partial Q/\partial P\cdot {\Delta }P+\partial Q/\partial {E}_{0}\cdot {\Delta }{E}_{0}=(-3.07)+(1.28)=-\,1.79\,{\rm{mm}}.$$

### Effects of the underlying surface change to streamflow in the Fen River watershed

Based on annual data collected from the Fen River watershed from 1951 to 1977, the mean streamflow in this area was 40.51 mm. The mean annual precipitation was 438.51 mm, and the mean long-term potential evapotranspiration was 955.11 mm. These data were used in Eq. (4), and the watershed characteristic parameter during the reference period was 2.7251. Similarly, based on annual data from 1978 to 2010, the mean streamflow in this area was 13.64 mm. The mean annual precipitation was 421.54 mm, and the mean long-term potential evapotranspiration was 931.84 mm. The watershed characteristic parameter during this reference period was 3.6635. The results are shown in Table 2.

**Table 2:** Watershed characteristic change in reference period and change period.

Period	*ω*
1951–1977	2.7251
1978–2010	3.6635
Change/Δ	0.9384

The impact amount caused by the watershed characteristic change to streamflow was calculated using the data in Table 2 as:

$${\Delta }{Q}_{watershed}=\partial Q/\partial \omega \cdot {\Delta }\omega =-\,25.42\,{\rm{mm}}.$$

### Streamflow variation reason analysis for the Fen River watershed

The potential causes of streamflow changes in the Fen River watershed are shown in Table 3. The results show that, the characteristic change in the watershed was the primary reason for streamflow change, and it led to a streamflow decrease of 25.42 mm. Changes in the watershed characteristics contributed 92.27% to streamflow changes from 1951 to 2010, while climate change only contributed 6.50% to streamflow changes.

**Table 3:** Streamflow variation reason analysis in research watershed.

	*∆Q*	*∆Q*_*climate*_	*∆Q*_*watershed*_	Error
Impact amount	−26.87 mm	−1.79 mm	−25.42 mm	0.34 mm
Contribution degree	100%	6.50%	92.27%	1.23%

### Double cumulative curve verification

The double cumulative curve method is the most simple, intuitive, extensive method in trend analysis, and it reflects long-term hydrological evolution. Its hypothesis is that precipitation changes are too slight to cause a change in watershed characteristics. Cumulative streamflow is impacted by both the underlaying characteristics of watersheds and climate change. Therefore, cumulative streamflow changes can be distinguished using the double cumulative curve method.

**Figure 5:**
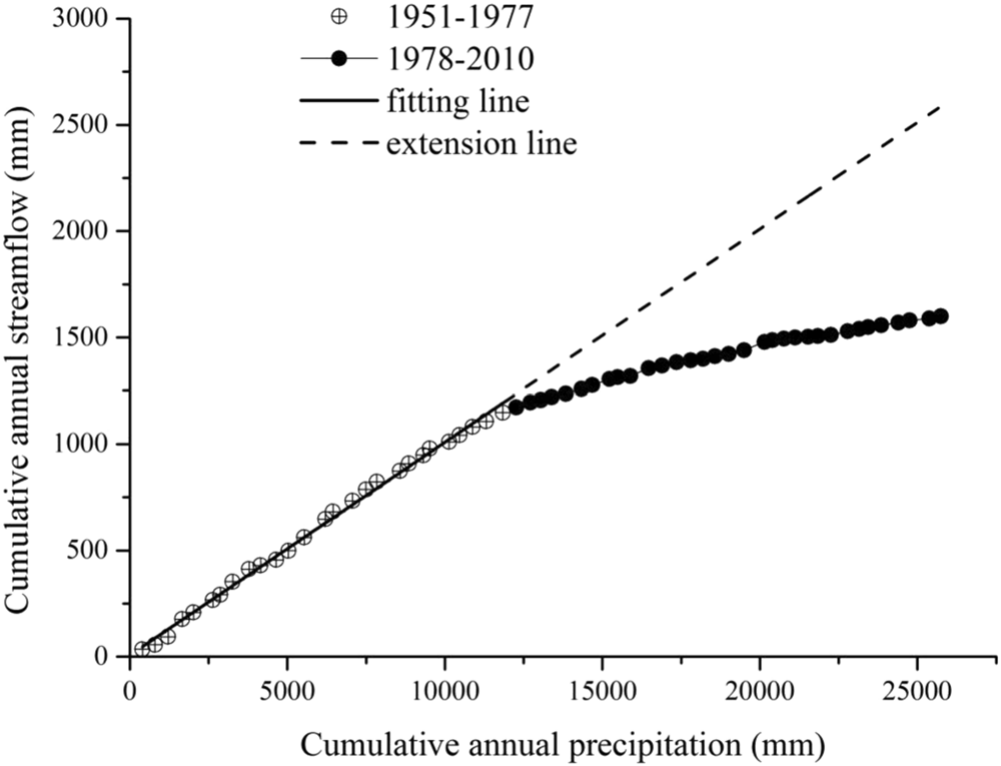
Double cumulative curves of precipitation and streamflow in the Fen River watershed.

Figure 5 shows the double cumulative curves of precipitation and streamflow for Fen River watershed. There is a significant linear relationship between the annual precipitation and cumulative streamflow. The slope of the fitted line changes significantly in 1978, this agrees with the Mann-Kendall test result. In addition, watershed streamflow was appears to be influenced by the underlaying characteristics. The distance between the deviation point and the extension line of the original fitting line represents the influence caused by the underlying surface changes.

Figure 5 also shows that the cumulative precipitation during the reference period was *Q* = 0.0967∑*P* + 19.4, and the determining coefficient were 0.9912. The results were obtained from long-term observations over a period of 60 years, and the statistical test had a 0.001 confidence level.

**Figure 6:**
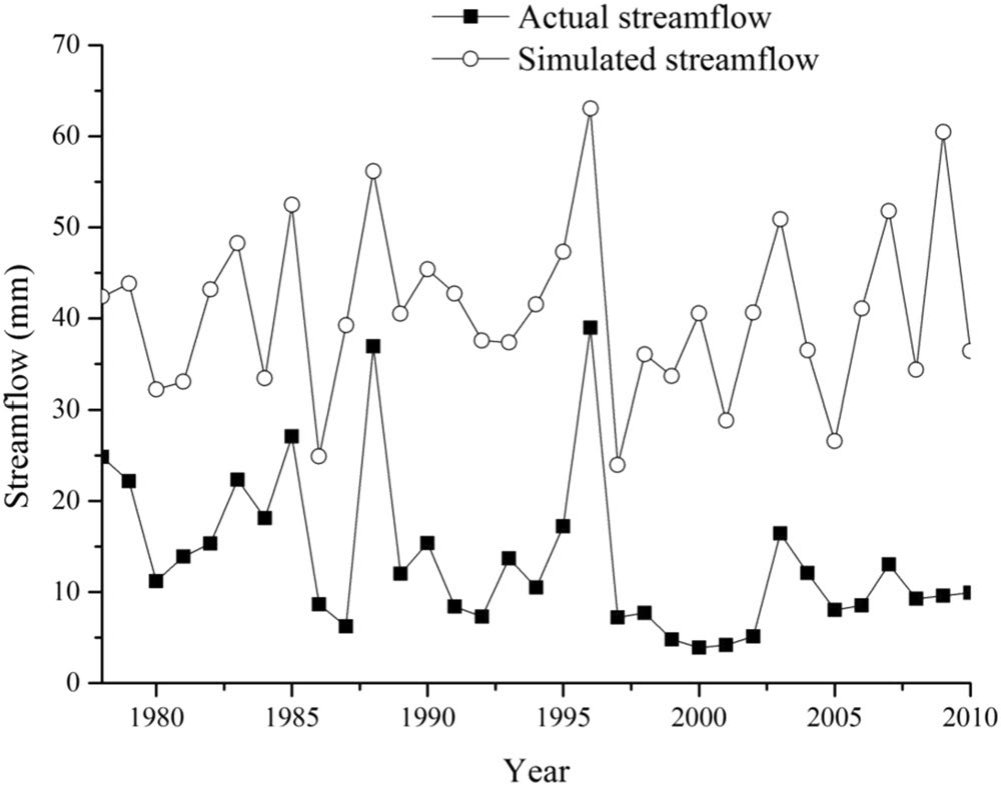
Double cumulative curve comparison of simulated value and observed values in change period.

The combined cumulative precipitation during the change period was calculated using the correlation equation, then the cumulative simulated streamflow was obtained. The cumulative watershed streamflow (Q′) during the change period could be inverted with this. Figure 6 shows a comparison of the double cumulative curve of the simulated values and of the observed values during the change period. The mean streamflow during the change period (1978–2010) was 13.64 mm, and the simulated value was 39.74 mm. Thus, the streamflow change amount (∆Qwatershed) caused by the underlaying surface characteristics was 26.09 mm. These results were close to the analysis results that used the Budyko hypothesis theory. Hence, the Budyko hypothesis theory made the results more credible.

## Discussion

### Attribution analysis

The Yellow River provides freshwater for approximately 107 million people, or approximately 8.7% of the total population in China. Many studies have indicated that the runoff of the Yellow River has decreased since the 1950s, and the studies have suggested that the runoff decrease of the Yellow River was the result of a decrease in precipitation and an increase in human activities. However, few studies have quantified the contribution of precipitation and human activities to runoff changes in the different sub-basins of the Yellow River^28^.

Previous studies^20,29–31^ have shown that the factors that affect streamflow can be summarized as climate change and human activities. Climate changes the circulation and distribution of water resources due to changes in temperature, radiation and wind speeds. Therefore, climate change has a direct impact on extreme hydrological events, such as droughts and floods. Human activities affect the process of regional water circulation by changing the type of the underlying surface, and there are many factors that affect runoff in a river basin, such as land use changes, water conservancy projects and water resources development. The Yellow River Basin is seriously affected by human activities. Most studies have shown that the impact of human activities on watershed runoff is much greater than that of climate change. In this study, the results show that the negative effects due to human activities were much larger than the effects of climate change with regards to streamflow changes in the Fen River watershed.

As a major tributary in the middle reach of the Yellow River, the Fen River is known for its high sediment yield. Since the 1950s, significant land use changes have taken place in the catchment to control soil erosion, maintain land productivity, and improve environmental quality. The extent and rate of these changes are unprecedented. These changes include tree plantations, establishment of pasturelands, building of terraces and sediment-trapping dams. Although these measures have reduced soil erosion, they have also resulted in noticeable changes in the streamflow regime. Given the range of the conservation measures, it is difficult to isolate the effects of individual measures on streamflow^32^.

Using hydrology data, this study confirmed that Fu’s equation is a valid framework for evaluating the effects of climate and changes in catchment characteristics on streamflow. More importantly, the relative roles of climate and underlying surface changes in the hydrological response were clarified. Based on Budyko-based coupled curves or equations, it was found that the hydrological responses were primarily driven by precipitation, potential evapotranspiration, and underlying surface changes. However, the relative contributions of climate and underlying surface changes to the hydrological responses have never been fully examined or quantified. Based on an attribution analysis of the Fen River watershed during 1951–2010, streamflow changes were primarily caused by changes in the underlying surface, which accounted for 92.27%, and changes in climate, which accounted for only 6.50%. These results indicate that underlying surface changes play a more important role in hydrological responses than climate does.

**Figure 7:**
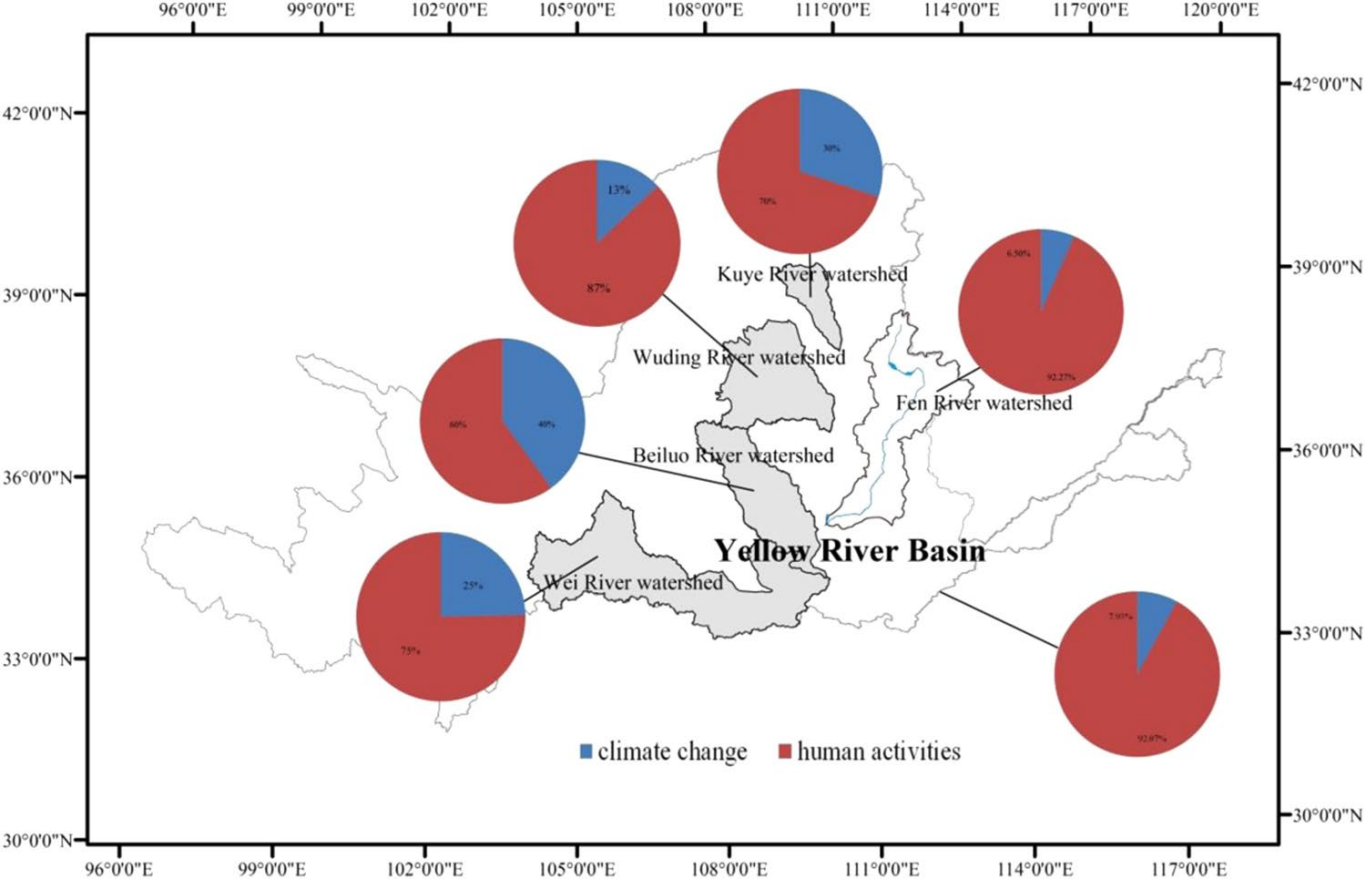
The map focusing on observed effects of the hydrological response in the Yellow River.

Several researchers^28^ have shown that the total contributions to runoff changes in the Yellow River were 7.93% from climate change, and 92.07% from human activities, and this is very similar to the results of this study. By comparing with other tributaries, some researchers have analyzed the attributes of runoff changes in the Kuye River watershed^33^, the Wuding River watershed^32^, the Beiluo River watershed^34^, and the Wei River watershed^35^. A map was developed that focuses on the observed hydrological responses, as seen in Fig. 7. In addition, these results suggest that human activities have been a dominant influencing factor in the runoff changes not only for each section, but also for the entire river basin.

### Cause-effects of mutation

As the results of Mann-Kendall mutation test, the streamflow of Fen River watershed showed a mutation in 1978, and it was similar with other rivers in Yellow River basin^32–35^. After studying climate change in the northern hemisphere and China, many researchers believe that three mutations occurred in the 1920s, 1960s and early 1980s^36^. Some researchers also believe that the abrupt change of global and Northern Hemisphere temperature in the past 100 years (1900–1990) is mainly a cold period before 1919, a warm period from 1920 to 1978, and a warmer period after 1979. The more obvious abrupt change years are 1920 and 1979^37^.

Apart from climate change, the impact of human activities seems to be a more important factor. In 1972, the Fenhe River was comprehensively harnessed, and gradually developed into the development of small and medium-sized projects in tributaries. And in 1970s, 2 large-scale reservoirs, 13 medium-sized reservoirs and nearly 40 small (1) reservoirs have been built in Fenhe River Basin. The total area of the basin is 12380 km^2^ and the total storage capacity is 1.306 billion m^3^.

## Conclusion

Based on the Budyko theory, the effects of climate and watershed characteristic changes on streamflow in the Fen River watershed were analyzed. The following results were concluded:

1. Annual streamflow of the Fen River watershed decreased significantly. There was a significant mutation point in 1978 when 1951–1977 was the reference period and 1978–2010 was the change period. The runoff amount decreased 26.87 mm (66.33%) during the change period compared to the reference period.
2. In the Fen River watershed, the sensitivity coefficients of runoff to precipitation, potential evapotranspiration and the watershed characteristic coefficient were 0.1809, −0.0551, −27.0882, respectively. These indicated that a 1 mm precipitation increase will increase runoff by 0.1809 mm; a 1 mm potential evapotranspiration increased will decrease runoff by 0.0551 mm; and a watershed characteristic factor change of 1 will lead to a runoff decrease of 27.0882 mm.
3. During 1951–2010, the watershed underlying surface change was the dominate factor in determining the runoff amount. It contributed 92.27% to the runoff amount change, whereas climate change only contributed 6.50% to the runoff change.
